# Osmolarity Controls Oscillatory Calcium Signaling to Reduce Autonomous Aldosterone Production in Zona Glomerulosa Cells

**DOI:** 10.1210/endocr/bqaf147

**Published:** 2025-10-14

**Authors:** Mohamed Diagne, Molly R Gerding, David T Breault, Edward H Nieh, Mark P Beenhakker, Paula Q Barrett, Nick A Guagliardo

**Affiliations:** Department of Stem Cell and Regenerative Biology, Harvard University, Cambridge, MA 02138, USA; Department of Pharmacology, University of Virginia, Charlottesville, VA 22908-0735, USA; Division of Endocrinology, Boston Children’s Hospital, Boston, MA 02115, USA; Department of Pediatrics, Harvard Medical School, Boston, MA 02115, USA; Broad Institute of MIT and Harvard, Cambridge, MA 02142, USA; Harvard Stem Cell Institute, Cambridge, MA 02138, USA; Department of Pharmacology, University of Virginia, Charlottesville, VA 22908-0735, USA; Department of Pharmacology, University of Virginia, Charlottesville, VA 22908-0735, USA; Department of Pharmacology, University of Virginia, Charlottesville, VA 22908-0735, USA; Department of Pharmacology, University of Virginia, Charlottesville, VA 22908-0735, USA

**Keywords:** aldosterone, primary aldosteronism, calcium oscillations, osmolarity, zona glomerulosa

## Abstract

Primary aldosteronism (PA) is characterized by autonomous aldosterone (Aldo) production, resulting in blood volume/electrolyte imbalance and hypertension. Intracellular calcium (Ca^2+^) is the principal signal driving Aldo synthesis in adrenal zona glomerulosa (zG) cells, and mutations in ion transport genes that regulate Ca^2+^ are frequently mediators of PA. When organized in intact rosette structures, zG cells are voltage oscillators; stimulation by angiotensin II (AngII) or loss of TWIK-related acid-sensitive potassium (TASK) channel function evokes stereotypic Ca^2+^ oscillations with bursting activity proportional to increased steroidogenesis. Here, we delineate the role of the osmolar-volume regulatory axis in the control of Ca^2+^ and Aldo production in adrenal slices. Strikingly, in both pharmacological and genetic models of PA, extracellular osmolarity (OSM_EC_) potently and reversibly regulated Aldo secretion and Ca^2+^ signaling. Elevated OSM_EC_ progressively suppressed Aldo production from AngII-stimulated adrenal slices and strongly inhibited autonomous production in both zG-specific TASK knockout slices and wild-type slices incubated with TASK inhibitors (TIs). To determine if the effects of OSM_EC_ on Ca^2+^ dynamics were causative, we imaged adrenal slices expressing zG-specific GCaMP6f incubated in variable osmotic media with TIs or AngII. Consistent with Aldo suppression, increasing osmolarity proportionally reduced the number of active cells and the Ca^2+^ activity of bursting cells evoked by TASK loss of function or AngII stimulation. Collectively, our findings identify OSM_EC_ as a broad regulator of zG excitability and adrenal steroidogenesis, and suggest that targeting volume-regulatory mechanisms such as the Na^+^-K^+^-2Cl^−^ cotransporter may offer a novel strategy to suppress Aldo autonomy in PA.

Primary aldosteronism (PA) is a hormonal disorder characterized by excessive production of aldosterone (Aldo) that is independent (autonomous) of the renin-angiotensin system (RAS). PA contributes significantly to hypertensive cardiovascular and renal disease and is highly prevalent—affecting 5% to 10% of individuals with hypertension and approximately 20% of those with resistant hypertension (1-3). The importance of early diagnosis and treatment is underscored by recent studies demonstrating that RAS-independent Aldo production is predictive of future adverse outcomes for preclinical, normotensive individuals (4-6).

Aldo synthesis in zona glomerulosa (zG) cells of the adrenal cortex is driven by intracellular calcium (Ca^2+^). Angiotensin II (AngII) and extracellular potassium (K^+^), major regulators of Aldo production, depolarize zG cells to increase the open probability of voltage-gated T-type (CaV3.2) and L-type (CaV1) Ca^2+^ channels (7). The resulting Ca^2+^ influx facilitates the multistep conversion of cholesterol to Aldo and the concomitant transcription of Aldo synthase (*CYP11B2*), which catalyzes the final rate-limiting step in synthesis (8). When assembled within their native rosette structures (9), zG cells respond to AngII and K^+^ by generating recurrent, large-amplitude (∼70 mV) membrane voltage oscillations (10). In turn, these prominent oscillations recruit several voltage-dependent conductances that further regulate Ca^2+^ entry. Notably, these oscillations are driven by voltage and not dependent on Ca^2+^ release from the endoplasmic reticulum (11). In adrenal slices expressing zG-specific GCaMP, a genetically encoded Ca^2+^ reporter, AngII evokes bursts of Ca^2+^ oscillations, the frequency of which correlates with Aldo output (11).

Mouse models of PA have shown dysregulation of intrinsic zG cell conductances is sufficient to drive autonomous Aldo production (12-24). For example, the global deletion of tandem-pore TWIK-related acid-sensitive K^+^ channel subunits (TASK-1 and TASK-3) depolarizes zG cells to produce autonomous Ca^2+^ oscillations and a prominent PA phenotype marked by elevated Aldo, low renin, and hypertension, effects that are not corrected by sodium (Na^2+^) loading or AngII type 1 receptor (AT1R) blockade (15, 16). Comparable effects result from the zG-specific deletion of TASK-1 and TASK-3 (zG-TASK-KO): robust, autonomous Ca^2+^ oscillations that drive steroidogenic activity in adrenal slices and produce a pronounced in vivo PA phenotype (19, 24, 25). Furthermore, a global gain-of-function mutation in the ClC-2 chloride (Cl^−^) channel (Clcn2R180Q/+)—initially identified in familial PA—evokes a mild PA phenotype (17, 20) compared to other PA models, whereas the global knockin of a constitutively open form of ClC-2 results in a marked increase in Cl⁻ efflux and a stronger zG depolarization to produce a severe PA phenotype (21). As with TASK channel loss of function, ClC-2 gain of function provokes Ca^2+^ oscillations in zG cells (21). Thus, targeting intrinsic depolarizing conductances within zG cells nested in native zG rosettes is sufficient to cause Ca^2+^ oscillations and Aldo autonomy.

Although strong evidence links ion channel mutations to increased Ca^2+^ influx within zG cells and, ultimately, PA (26, 27), direct evidence linking such mutations to decreased Ca^2+^ influx within zG cells and hypoaldosteronism is lacking. Nevertheless, early studies in canine adrenals by Schneider and colleagues (28-30) demonstrated that adjusting extracellular osmolarity (OSM_EC_) is sufficient to modulate Aldo secretion evoked by K⁺ or AngII. Similar effects have been observed in isolated rat zG cells (31). Yet, it remains unclear if or how OSM_EC_ regulates the coordinated oscillatory activity of zG cells to affect Aldo production. Here, we manipulate OSM_EC_ to resolve whether cell volume-regulatory mechanisms can be leveraged to suppress oscillatory Ca^2+^ signaling and restrain autonomous Aldo production associated with PA.

## Materials and Methods

### Mice

Experiments were conducted using mice aged 45 to 90 days, housed in a temperature- and humidity-controlled vivarium under a 12:12-hour light:dark cycle until the day of the experiment. Adrenal slices were prepared from mice with wild-type (WT) TASK-1 and TASK-3 (AS⁺^/^⁺::TASK-1⁺^/^⁺::TASK-3⁺^/^⁺) and zG-specific TASK deletion (zG-TASK-KO; AS⁺^/Cre^::TASK-1^fl/fl^::TASK-3^fl/fl^) (11, 24, 32). Depending on the experimental application, slices were prepared with or without Cre-dependent expression of the genetically encoded Ca^2+^ indicator GCaMP6f for Ca^2+^ imaging or Aldo secretion assays, respectively, as previously described (11, 24). To account for sex as a biological variable, experiments were conducted both in male and female mice, yielding consistent findings across groups. For in situ RNAscope experiments, mTmG mice (The Jackson Laboratory, 007676, B6.129(Cg)-*Gt(ROSA)26Sor^tm4(ACTB-tdTomato,-EGFP)Luo^*/J) were crossed with AS⁺^/Cre^ to generate an AS⁺/^Cre^::mTmG^+/−^ mouse line, as previously reported (32).

### Acute Adrenal Slice Preparation

Adrenal glands were collected from deeply anesthetized mice the day of the experiment (ketamine, 10-15 mg, intraperitoneally), carefully dissected free of surrounding adipose tissue, and embedded in 3.2% agar prepared in PIPES buffer. Embedded glands were sectioned into 60- to 70-μm slices using a vibratome (Microslicer Zero-1, Ted Pella) in ice-cold PIPES-based incubation buffer containing (in mM): 20 PIPES, 117 NaCl, 3 KCl, 1 CaCl_2_, 1 MgCl_2_, 25 D-glucose, and 5 NaHCO_3_, adjusted to pH 7.3 with 10 N NaOH. Slices were subsequently incubated at 37 °C for 25 minutes prior to experimental use.

### Aldosterone Secretion

Culture media was formulated using Dulbecco’s modified Eagle’s medium/Nutrient Mixture F-12 Ham powder (with 15 mM HEPES; L-glutamine-, CaCl_2_-, MgCl_2_-, MgSO_4_-, and NaHCO_3_-free; Millipore Sigma, D9785) supplemented with 15 mM NaHCO_3_, 0.1% bovine serum albumin, 2.5% Nu-Serum IV (Corning, 355104), 200 µM L-alanyl-L-glutamine dipeptide (GlutaMAX, Gibco, 35050-061), and 1× Insulin-Transferrin-Selenium (Gibco, 41400-045). Final ionic concentrations were adjusted to (in mM): 4.1 KCl, 2 CaCl_2_, 110 NaCl, 0.6 MgCl_2_, 0.4 MgSO_4_. The pH was titrated to 7.3 using 10 N NaOH. Osmolarity was set to 260 or 280 mOsm by dilution with deionized water containing electrolytes ([in mM]: 4 KCl, 2 CaCl_2_, 0.6 MgCl_2_, and 0.4 MgSO_4_) to maintain consistent membrane potential-relevant ion concentrations. Sucrose was added to 280 mOsm media to generate 295 to 325 mOsm media without altering the ionic composition. Final osmolarity was determined using a vapor pressure osmometer (VAPRO 5600, ELITechGroup).

Adrenal slices were cultured on polytetrafluoroethylene membranes (Millicell, PICM03050), placed in 12-well plates (one slice per well), and maintained at 37 °C in a humidified incubator with 5% CO_2_. Slices were preincubated in undiluted culture media (400 µL) for 2 hours prior to treatment. Media was collected after a 30-minute baseline period (310 mOsm), followed by transfer to variable osmotic media containing either vehicle (control), AngII (50, 300, 500, or 1000 pM; Bachem, H-1705), or TASK inhibitors (TIs) (200 nM A1899, Tocris, 6972; and 200 nM PK-THPP, Tocris, 5338) with or without 20 µM furosemide (Tocris, 3109) for an additional 30-minute treatment period. Media samples were collected at the end of each phase for Aldo quantification.

Aldo levels were measured using a radioimmunoassay (Tecan US Inc, MG13051; RRID: AB_3712182) and reported as picogram per slice per hour. Because the number of functional zG cells per slice was not directly quantified, total Aldo per slice was treated as an approximate measure. To account for interslice variability, a within-well normalization strategy was employed: Fold change in Aldo secretion was calculated for each slice by dividing posttreatment values by baseline values. For each animal, the mean fold change from vehicle-treated control slices was used to normalize all treatment conditions.

### GCaMP6f Ca^2+^ Imaging

Adrenal slices were stabilized in an ultraquiet chamber (Warner Instruments) using a friction-fit nylon harp to minimize movement during acquisition as previously described (19) using a Zeiss Axio-Examiner microscope equipped with a 488 nM wavelength X-Cite XLED light source and a 63× dipping objective. Images were acquired continuously at 10 Hz for up to 25 minutes using a Hamamatsu ORCA-Flash 4.0 sCMOS camera controlled by SlideBook 6 software (Intelligent Imaging Innovations, 3i). Samples were imaged under low-light conditions using 2 neutral density filters in series to minimize photobleaching and phototoxicity; light intensity was attenuated by both the software-controlled neutral density module within the light source and a discrete Zeiss fluorescence attenuator (Zeiss, 423730-9130), which reduced illumination by approximately 90% and 98%, respectively. To compensate for the reduced signal under low-light conditions, images were binned 4 × 4 during acquisition to enhance signal-to-noise ratio. All image sequences were saved as high-quality multipage TIFF files for subsequent analysis.

Adrenal slices were continuously perfused with PIPES-based imaging buffer. For 280 mOsm experiments, the imaging buffer contained (in mM): 20 PIPES, 104 NaCl, 4 KCl, 2 CaCl_2_, 1 MgCl_2_, and 25 D-glucose, adjusted to pH 7.3 with 10 N NaOH. To prepare 310 mOsm solution, sucrose was added to the 280 mOsm buffer while keeping all other components unchanged. For both AngII and TI experiments, slices were preincubated for 10 minutes with imaging buffer containing either AngII (500 pM or 1 nM) or a combination of 200 nM A1899 and 200 nM PK-THPP immediately prior to imaging. These compounds were also included in the perfusate throughout the duration of each experiment.

In some experiments, adrenal slices were initially perfused with either 280 or 310 mOsm imaging buffer for 10 minutes, after which the perfusate was switched to the opposite osmolarity (280 → 310 or 310 → 280), followed by a 5-minute equilibration period and a second 10-minute imaging session (“osmolar switch” experiments). The second imaging period was analyzed independently, as continuous region of interest (ROI) tracking was often not feasible following the osmolarity switch, which caused shifts in the focal plane.

### Image Analysis of Ca^2+^ Oscillations

Ca^2+^ imaging data were processed using a multistep pipeline to ensure accurate motion correction, background subtraction, ROI detection, and signal validation (Supplementary Fig. S1) (33). After image acquisition, raw multipage TIFF image sequences acquired with SlideBook software were motion-corrected using the Mesmerize software suite (34). Following motion correction, rolling background subtraction was applied using ImageJ to reduce low-frequency background fluorescence. ROI detection and signal extraction were performed using Suite2p (https://github.com/MouseLand/suite2p), which included automated segmentation (ROIs, Supplementary Fig. S2) (33), and subsequent signal extraction and deconvolution of fluorescence traces for each ROI (Supplementary Fig. S3) (35). In cases in which ROI profiles overlapped, signals were extracted exclusively from the nonoverlapping regions. We used Suite2p's neuropil detection algorithm—typically used to identify axons and dendrites surrounding somata in neuronal cell populations—to mitigate scattered light inherent in widefield fluorescence imaging of tissue slices, and potential contamination from intensely fluorescent adjacent ROIs. This algorithm was repurposed to subtract background contamination from the raw traces, thereby enhancing signal specificity (Supplementary Fig. S3A-S3D) (33).

Ca^2+^ transient detection was performed using a custom MATLAB peak detection script (Supplementary Fig. S3E-S3G) (33, https://github.com/NickGuag/detectORAMA.git). ROIs were first filtered based on signal quality by computing the minimum and maximum SD within a moving window; ROIs with a maximum:minimum SD ratio exceeding a defined threshold were excluded due to poor signal-to-noise. Transients were identified from deconvolved fluorescence traces (Supplementary Fig. S3F) (33) by applying dual thresholds for each nonzero value: (1) a percentage of the maximum deconvolved trace and (2) minimum peak amplitude of the corresponding background-subtracted fluorescence relative to baseline noise. Threshold values were manually adjusted for individual ROIs to optimize detection sensitivity. Transient peaks were identified by aligning the threshold-filtered deconvoluted signal to the maxima of the fluorescence signal within a ± 5 frame window (ie, ± 0.5 seconds; Supplementary Fig. S3G and S3H) (33). A final moving filter was applied to remove minor fluctuations superimposed on larger transients, improving the specificity of transient detection.

To validate detected transients and minimize false-positive transients, we incorporated components of the SEUDO package (https://github.com/adamshch/SEUDO) (36). Specifically, we used the transient detection module (classifyTransients function) to identify low-correlation and high-noise transients, which compares the detected transients with the background-subtracted images for efficient artifact rejection of spurious signals.

Bursting activity was analyzed as previously described (11). Ca^2+^ transient interpeak interval distributions were modeled as a mixture of gaussian and exponential components, and the intersection point of these distributions was used to define the maximum interpeak interval within a burst. In pilot experiments, this threshold was determined to be 5.6 and 5.3 seconds for TI and AngII, respectively. Transients were classified as part of a burst if at least 3 consecutive events occurred with intertransient intervals all below this threshold, and transient periods were calculated from interpeak intervals within a burst. This approach allowed for consistent identification of temporally clustered activity (ie, Ca^2+^ bursts).

To quantify cellular activity, the number of active ROIs was normalized to the zG area, yielding a density measure of active cells per µm^2^. zG area was delineated and calculated using ImageJ software.

### In Situ RNAscope

Adult AS⁺/^Cre^::mTmG^+/−^ mice that express GFP under the control of the *Cyp11b2* promoter were deeply anesthetized and transcardially perfused with phosphate-buffered saline, followed by 4% paraformaldehyde in phosphate-buffered saline. Adrenal glands were harvested, post-fixed in 4% paraformaldehyde overnight, and sectioned at 50 µm for analysis. Multiplexed RNA in situ hybridization (RNAscope; Advanced Cell Diagnostics) was performed on adrenal slices to detect transcripts encoding the Na⁺-K⁺-2Cl⁻ cotransporter NKCC1 (*Slc12a2,* Advanced Cell Diagnostics, catalog No. 311911-C2) or the endothelial cell marker PECAM-1 (*Pecam1,* Advanced Cell Diagnostics, catalog No. 316721-C3) labeled with far-red TSA Vivid 650 fluorophore kit (Advanced Cell Diagnostics, catalog No. 323273) following the manufacturer's protocol (https://acdbio.com/referenceguide). Fluorescence z-stack images were acquired using confocal microscopy with a 63× objective to detect GFP (*Cyp11b2*+ cells), tdTomato *(Cyp11b2*- cells*),* and TSA Vivid 650 fluorophore *(Slc12a2 or Pecam1* probes). Images are presented after linear contrast adjustment. *Slc12a2* messenger RNA expression was evaluated qualitatively based on presence or absence of label, using *Pecam1* as a negative control, without quantitative measurement or statistical analysis.

### Statistics

All statistical analyses were performed using GraphPad Prism software (version 10.4.2; GraphPad Software Inc). Data are presented as mean ± SEM. For Aldo secretion assays, values represent the average Aldo produced by 2 or 3 replicate slices per condition from a distinct mouse. For Ca^2+^ imaging experiments, data represent the mean ± SEM of ROIs within a slice. Each mouse contributed a single data point per condition, except for AngII Ca^2+^ imaging experiments, in which some animals contributed at most 2 data points per experimental condition. Data from male and female mice were pooled for statistical analysis. Sex-specific data points are distinguished in the graphs using different symbols, as described in the figure legends. Comparisons between 2 groups were made using 2-tailed unpaired or paired *t* tests, and 3 or more comparisons analyzed by 1- or 2-way analysis of variance, as appropriate. A *P* value of less than .05 was considered statistically significant. Detailed statistical methods for each analysis are provided in the figure legends.

### Study Approval

All procedures were performed in accordance with the National Institutes of Health Guide for the Care and Use of Laboratory Animals and were approved by the University of Virginia Institutional Animal Care and Use Committee.

## Results

**Figure 1.**
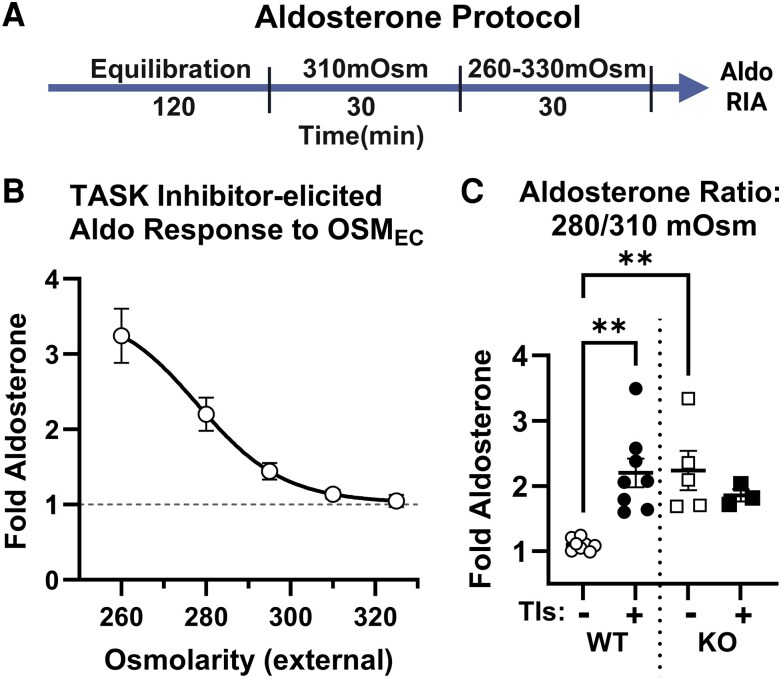
Regulation of acute aldosterone (Aldo) production by extracellular osmolarity (OSM_EC_) in adrenal slices. A, Schematic of Aldo production protocol generated with Biorender.com. B, Fold change in Aldo over baseline production (310 mOsm) elicited by TWIK-related acid-sensitive potassium (TASK) inhibitors (TIs; 200 nM A1899 and 200 nM PK-THPP) in wild-type (WT) slices over a range of OSM_EC_ (260-330 mOsm). Data were fit with a nonlinear (sigmoidal) regression; *R^2^* = 0.726. C, Fold change in Aldo over baseline (310 mOsm) in adrenal slices from WT (left) and zona glomerulosa (zG)-TASK–knockout (KO) (right) mice in 280 mOsm media with either TIs (+) or vehicle (−) treatment. TIs: 200 nM A1899 and 200 nM PK-THPP. Data are shown as mean ± SEM. B, n = 4 mice; C, N = 8 WT/5 KO mice. Statistical significance in (C) was determined using one-way analysis of variance and Tukey correction for multiple comparisons; **P less than .001.

We first evaluated the effects of OSM_EC_ on Aldo production using a pharmacological model of PA that employed the combined inhibition of TASK-1 and TASK-3 ion channels (TIs, 200 nM A1899 and 200 nM PK-THPP) in acute adrenal slices prepared from WT mice. Aldo concentrations were measured in the culture media at 30-minute intervals and normalized to baseline secretion prior to TASK inhibition (Fig. 1A). Elevated OSM_EC_ was achieved by adding sucrose to the media, keeping the concentrations of all other media electrolytes equivalent with osmolarity adjustment. Across a range of OSM_EC_ values (260-325 mOsm), increasing OSM_EC_ progressively suppressed TI-induced autonomous Aldo production (Fig. 1B), with Aldo levels in 310 and 325 mOsm media comparable to unstimulated baseline. Aldo production in 280 mOsm media (fold increase: WT + TIs: ∼2.2, zG-TASK-KO: ∼2.2; Fig. 1C) closely mirrored the in vivo physiological response of zG-TASK-KO mice under conditions of RAS suppression, either on a high Na^+^ diet (∼2.5-fold Aldo increase) or with AT1R antagonist candesartan (∼2.0-fold Aldo increase) treatment (19). Consequently, in subsequent experiments we selected 280 mOsm as representing the physiological condition and used 310 mOsm media to assess the potential for osmolarity-dependent suppression of Aldo production. We observed that reducing the osmolarity from 310 (suppressed) to 280 mOsm permitted TI stimulation of Aldo production in WT adrenal slices, whereas lowering OSM_EC_ alone (in the absence of TIs) did not affect Aldo output (Fig. 1C, left). Thus, OSM_EC_ functions as a modulator of stimulus-elicited Aldo production, but a 30 mOsm reduction in media osmolarity alone was insufficient to alter Aldo output.

To assess if elevated OSM_EC_ was also effective in reducing Aldo production in a genetic loss-of-function mouse model of PA, we tested adrenal slices from zG-TASK-KO mice. In zG-TASK-KO slices, Aldo production was significantly higher than that of unstimulated WT slices in 280 mOsm, but not 310 mOsm, media (Supplementary Fig. S4) (33). The ratio of Aldo produced from each slice at 280 vs 310 mOsm media, a comparative metric of Aldo suppression, was also similar between TASK channel dysfunction elicited pharmacologically and genetically (Fig. 1C). Importantly, pharmacological inhibition of TASK-1/TASK-3 had no effect on Aldo production in zG-TASK-KO slices, supporting selectivity of the TIs (see Fig. 1C, right). Collectively, these findings demonstrate that OSM_EC_ effectively regulates both pharmacological and genetic models of PA, wherein elevated OSM_EC_ suppresses autonomous Aldo production.

**Figure 2.**
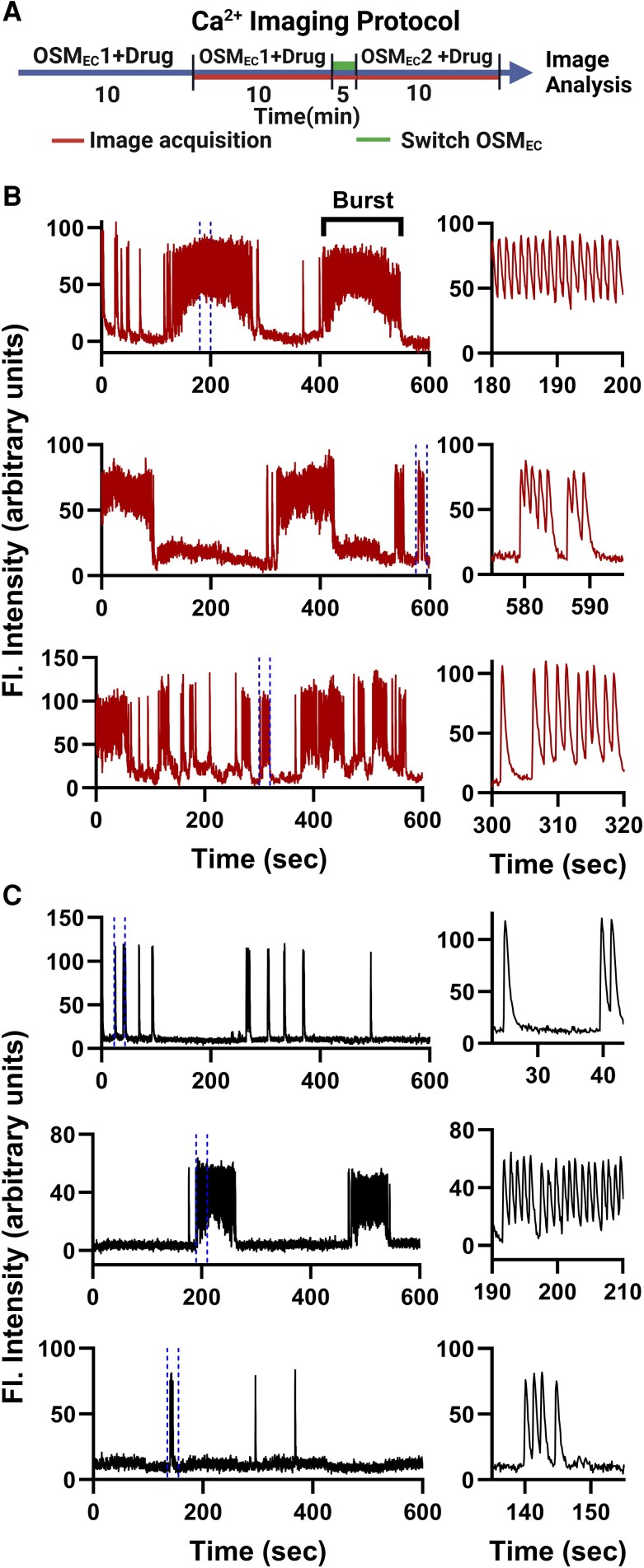
Representative fluorescence traces from individual regions of interest in adrenal slices incubated with TWIK-related acid-sensitive potassium (TASK) inhibitors (TIs) under different osmolarity conditions. A, Schematic of Ca^2+^ imaging protocol generated with Biorender.com. B, Slices were preincubated in either B, 280 mOsm or C, 310 mOsm PIPES-buffered media containing TIs (200 nM A1899 and 200 nM PK-THPP) for 10 minutes, followed by 10 minutes of continuous imaging. Bursts were defined as 3 or more consecutive fluorescence peaks with interpeak intervals less than 5.6 seconds. Brackets (B, top) indicate an example burst. Left panels: full 10-minute recording; right panels: 20-second segment shown on an expanded time scale corresponding to the region between the blue dotted lines in the respective left panel.

To determine whether OSM_EC_ concurrently regulates oscillatory Ca^2+^ signaling, we generated WT mice with zG-specific expression of the genetically encoded Ca^2+^ indicator GCaMP6f. Compared to GCaMP3, the indicator used in earlier studies (11, 24), GCaMP6f provides improved sensitivity and a higher signal-to-noise ratio, enabling the use of low-intensity fluorescence for extended imaging sessions (>25 minutes) without signal degradation. Acute adrenal slices were preincubated in either 280 or 310 mOsm PIPES-buffered solution containing TIs for 10 minutes prior to imaging. Images were captured under continuous perfusion using widefield fluorescence microscopy for up to 25 minutes, with TIs included in the perfusion buffer throughout the experiment (Fig. 2A). Fig. 2B and 2C shows representative fluorescence traces of Ca^2+^ transients (ie, spikes of intracellular Ca^2+^) elicited by TIs, which frequently occurred in series as bursts, features characteristic of oscillatory activity. In 280 mOsm media (ie, Aldo permissive), zG Ca^2+^ transients were robust and often organized into prolonged bursts (Fig. 2B, movie 1) (33). By contrast, zG cells perfused in 310 mOsm media (ie, Aldo suppressive) generated fewer bursts that were shorter in duration (Fig. 2C, movie 2) (33).

**Figure 3.**
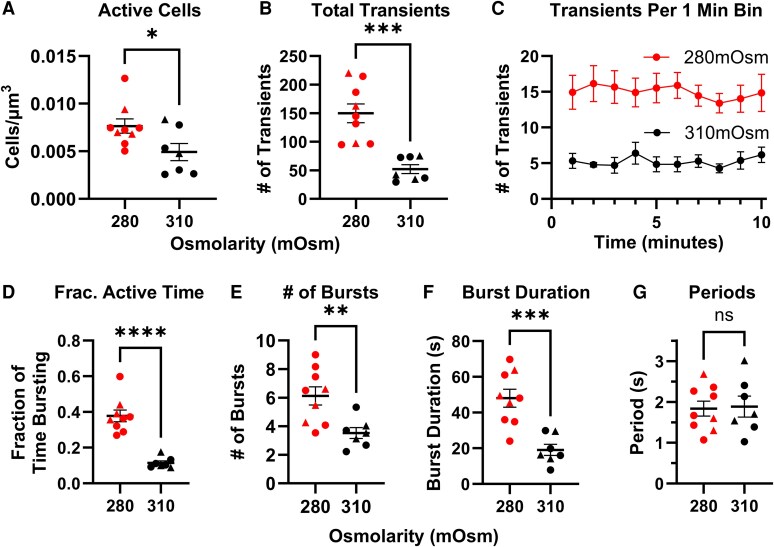
Quantification of Ca^2+^ activity in adrenal slices incubated with TWIK-related acid-sensitive potassium (TASK) inhibitors (TIs, 200 nM A1899 and 200 nM PK-THPP) in 280 mOsm and 310 mOsm media. A, Number of active cells per zona glomerulosa (zG) area, defined as regions of interest (ROIs) with 3 or more Ca^2+^ transients elicited during the 10-minute imaging period. B, Total number of Ca^2+^ transients per slice. C, Mean steady-state Ca^2+^ transient frequency in 1-minute bins over the 10-minute imaging time course for each extracellular osmolarity (OSM_EC_) condition, demonstrating stable responsiveness and minimal signal degradation. D, Fraction of time spent in active Ca^2+^ bursting. E, Number of Ca^2+^ bursts and F, burst duration per ROI. G, The intraburst Ca^2+^ transient period, as defined by the interpeak interval. Data represent mean ± SEM of activity within ROIs from each adrenal slice; A, B, D to G, Slices from male mice are indicated with circles, and female mice indicated with triangles. N = 9 mice. Statistical significance was determined using 2-tailed *t* test; *P less than .05; **P less than .01; ***P less than .001; ****P less than .0001.

To quantify this oscillatory activity, we developed a custom analysis pipeline (see Supplementary Fig. S1) (33) incorporating Mesmerize software (for motion correction), Suite2p software (for ROI detection and signal deconvolution), custom MATLAB scripts (for Ca^2+^ transient detection), and Seudo software (for Ca^2+^ transient validation). Notably, the first 10 minutes of each recording revealed that 280 mOsm activated a greater number of zG cells per area—as defined by ROIs exhibiting 3 or more Ca^2+^ transients—and a greater total number of Ca^2+^ transients per cell relative to recordings performed in 310 mOsm (Fig. 3A and 3B). Importantly, transient frequency remained stable over time within each osmolarity condition, indicating minimal signal degradation and consistent cellular responsiveness (Fig. 3C), even under 310 mOsm conditions.

We previously demonstrated that discrete oscillatory Ca^2+^ bursts serve as a key unit of Ca^2+^ signaling activity among active zG cells that correlates with Aldo production (11). Here, quantifying burst frequency and duration revealed that zG cells perfused in 280 mOsm permissive media burst more robustly (Fig. 3D) during TASK channel inhibition, indicated by a greater number of Ca^2+^ bursts (Fig. 3E), but also by longer burst durations (Fig. 3F), compared to 310 mOsm–suppressive media. Interestingly, the observed decrease in burst duration evoked by TASK channel inhibition by 310 mOsm media contrasts with our previously observed responses evoked by AngII stimulation (11), wherein burst number but not duration changes with AngII concentration. Nonetheless, consistent with the response across AngII doses (11), the intertransient period of Ca^2+^ oscillations within a burst remained invariant across osmolarity conditions (Fig. 3G).

To assess the reversibility of OSM_EC_ actions on TI-evoked stimulation, we compared steady-state Ca^2+^ activity during the first and last 10 minutes of each imaging experiment. After the first 10 minutes of imaging, the perfusate osmolarity was switched (osmolarity switch, ie, 280-310 mOsm or vice versa) and imaged for an additional 15-minute period (Fig. 2A). The 5-minute transition period after osmolarity switch was excluded from analysis. Because OSM_EC_-induced cell volume changes can shift the imaging plane, the final 10-minute imaging period was not analyzed on the same subset of cells (see “Materials and Methods”) and in some cases discarded due to loss of focal clarity.

**Figure 4.**
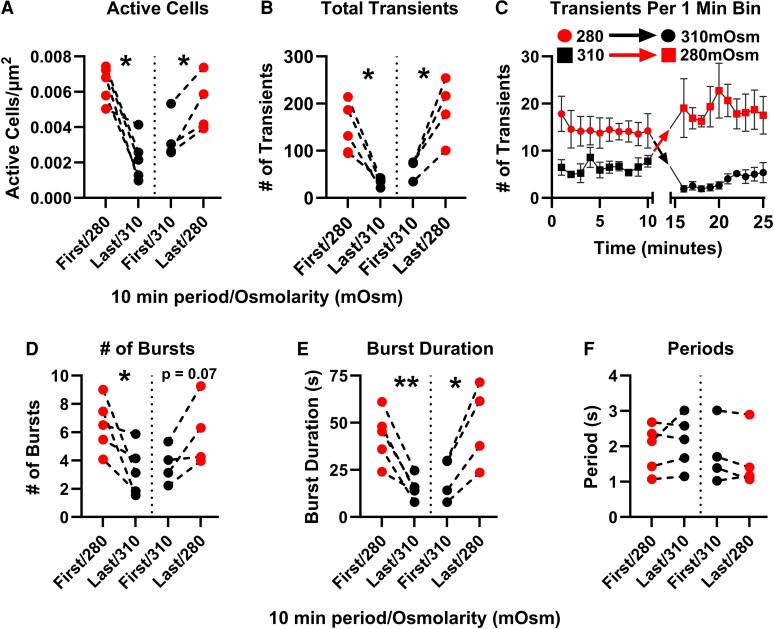
Within-slice analysis of Ca^2+^ activity elicited by TWIK-related acid-sensitive potassium (TASK) inhibitors (TIs) following osmolarity switching. Adrenal slices were imaged for 10 minutes under initial osmolarity conditions (either 280 or 310 mOsm), followed by a 5-minute equilibration period and a subsequent 10-minute imaging session after switching to the opposite osmolarity. A, Number of active cells per zona glomerulosa area before and after the osmolarity switch. B and C, Total number of Ca^2+^ transients per slice, showing a decrease on switching from 280 to 310 mOsm and an increase from 310 to 280 mOsm. D, Number of Ca^2+^ bursts per slice and E, burst duration were reduced in 310 mOsm buffer solution. F, Interburst period, which remained unchanged across conditions. Data pairs represent mean ± SEM of all regions of interest of a single adrenal slice. N = 5 mice. Statistical significance was determined using 2-tailed paired *t* test. *P less than .05; **P less than .01.

As shown in Fig. 4, osmolarity shifts from 280 to 310 mOsm resulted in a significant reduction in zG cell Ca^2+^ activity, as can be seen in movie 3 (33). OSM_EC_ of 310 mOsm decreased both the number of active zG cells (Fig. 4A) and the mean Ca^2+^ activity of active zG cells, as measured by the total number of Ca^2+^ transients (Fig. 4B and 4C). Conversely, switching from 310 to 280 mOsm permitted an increase in both activity metrics (movies 4 and 5) (33), in agreement with the changes in activity observed in between-slice comparisons (Fig. 3A and 3B). Similarly, the mean number of Ca^2+^ bursts (Fig. 4D) and the mean burst duration (Fig. 4E) decreased in 280 → 310 mOsm and increased in 310 → 280 mOsm during the final 10-minute imaging period. By contrast, intraburst Ca^2+^ transient periods remained unchanged across osmotic conditions (Fig. 4F), further supporting the observation that OSM_EC_ modulates the number and duration of Ca^2+^ oscillatory activity without altering their transient periodicity. These within-slice analyses confirm that the effects of OSM_EC_ on Aldo-associated Ca^2+^ dynamics are both robust and reversible and are not attributable to slice-to-slice variability.

**Figure 5.**
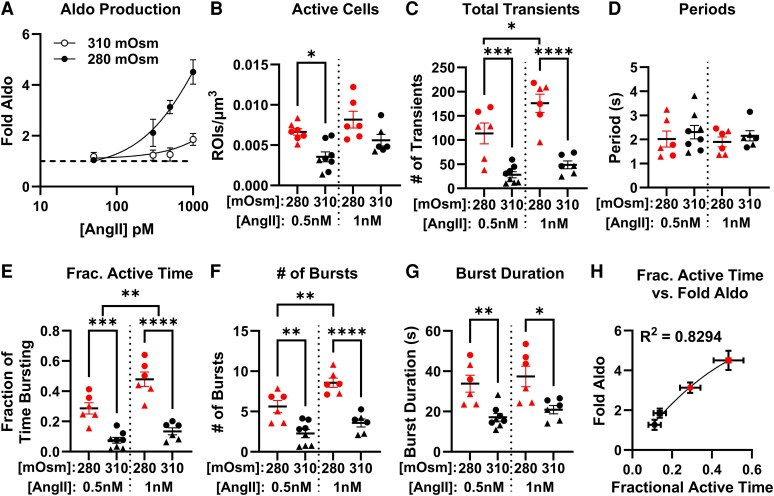
Effects of extracellular osmolarity (OSM_EC_) on angiotensin II (AngII)-evoked aldosterone (Aldo) secretion and Ca^2+^ signaling. A, Aldo production in adrenal slices exposed to increasing concentrations of AngII (50 pM, 300 pM, 500 pM, and 1 nM) under varying osmolarity conditions (280 and 310 mOsm). At each AngII concentration, Aldo output was inversely correlated with OSM_EC_. B, Quantification of the number of active cells/µm^2^ in response to 500 pM and 1 nM AngII under 280 and 310 mOsm conditions. C, Total number of Ca^2+^ transients per slice in response to 500 pM and 1 nM AngII at 280 and 310 mOsm. D, Intraburst transient periods remained unchanged across osmolarity conditions. E, Fraction of time spent in active Ca^2+^ bursting. F, Number of Ca^2+^ bursts per slice and G, burst duration. H, Correlation between mean Aldo production (from Fig. 5A) and fractional active time (from Fig. 5E), across all 4 conditions (500 pM and 1 nM Ang II in both 280 and 310 mOsm media). Line indicates a nonlinear fit of data; *R^2^* = 0.8294. Data represent mean ± SEM of all activities within regions of interest from a single adrenal slice. Slices from male mice are indicated with circles, and female mice indicated with triangles. N = 6-8 mice per condition. Statistical significance was determined using 2-way analysis of variance with Šídák multiple comparisons test. *P less than .05; **P less than .01; ***P less than .001; ****P less than .0001.

To determine whether OSM_EC_ exclusively modulates Ca^2+^ signaling and steroidogenesis in the context of PA-associated autonomy, we next evaluated the effects of osmolarity on nonautonomous, AngII-evoked Aldo production. Acute adrenal slices were cotreated with increasing concentrations of AngII (50 pM, 300 pM, 500 pM, and 1 nM) in media of varying osmolarities (280 and 310 mOsm). At each AngII concentration, Aldo production was inversely correlated with osmolarity, with lower OSM_EC_ media permitting higher Aldo output (Fig. 5A), consistent with zG responses in our pharmacological PA model (Fig. 1B). Thus, OSM_EC_ broadly regulates Aldo production.

To determine if OSM_EC_ produced similar changes in AngII-evoked Ca^2+^ dynamics, we performed imaging experiments using 500 pM and 1 nM AngII under either 280 (permissive) or 310 mOsm (suppressive) conditions. Consistent with the effects observed during TASK channel inhibition, 310 mOsm reduced the number of active cells at 500 pM AngII compared to 280 mOsm buffer, with a similar trend at 1 nM Ang (*P* = .089; Fig. 5B). As observed with TI-induced autonomy, 310 mOsm reduced the mean number of Ca^2+^ transients per active cell compared to 280 mOsm (Fig. 5C), without changing the intertransient period (Fig. 5D), resulting in a decrease in the fraction of time zG cells spend bursting (Fig. 5E). Both a reduction in the number of Ca^2+^ bursts (Fig. 5F), as well as a reduction in the burst duration (Fig. 5G), accounted for the 310 mOsm–induced reduction in fractional active time. Interestingly, this increase in burst duration contrasts with the dose-dependent response to AngII stimulation wherein burst number increases with concentration but burst duration remains constant (11) (see Fig. 5F and 5G), suggesting only a partial overlap in the mechanisms underlying AngII stimulation and OSM_EC_ modulation. These results replicate the OSM_EC_-induced changes elicited during PA-simulation and highlight the unique modulation of burst duration by OSM_EC_ regardless of the mode of stimulation (ie, channel loss of function or secretagogue). In Fig. 5H, we plotted mean Aldo production (from data shown in Fig. 5A) against fractional active time (see Fig. 5E)—which integrates both burst number and duration—across all 4 conditions (500 pM and 1 nM AngII in both 280 and 310 mOsm media). Consistent with our previous findings (11), these variables were strongly correlated, as indicated by a nonlinear fit (*R^2^* = 0.83), confirming that Ca^2+^ burst activity serves as a reliable proxy for Aldo output. Together, these findings demonstrate that OSM_EC_ modulates both Aldo secretion and the underlying Ca^2+^ signaling in response to both pharmacological/pathological and physiological stimuli, highlighting the importance of Ca^2+^ burst dynamics as a mediator of osmotic regulation of adrenal steroidogenesis.

**Figure 6.**
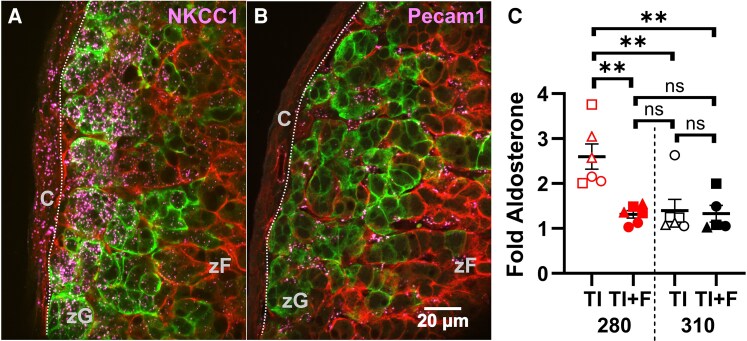
NKCC1 inhibition normalizes TWIK-related acid-sensitive potassium (TASK) inhibitor (TI)-induced aldosterone production in adrenal slices. A and B, In situ imaging of mouse mRNA. Puncta (Cy5, magenta) report mRNA expression for A, *Slc12a2* (NKCC1) and B, *Pecam1* (PECAM-1). Membrane-GFP (green) indicates *Cyp11b2*+ Cre expression. Data are presented as mean ± SEM fold increase in aldosterone (Aldo) production by slices treated with TIs (200 nM A1899 and 200 nM PK-THPP) with or without 20 µM furosemide (TI + F) at either 280 or 310 mOsm for 30 minutes (data separated by vertical dotted line) and normalized to previous 30-minute unstimulated Aldo production in 310 mOsm media. Two slices were analyzed from each of 3 mice, with individual mice represented by different symbols (triangle, square, or circle). C, Adrenal capsule; zF, zona fasciculata; zG, zona glomerulosa; dotted line approximates boundary between capsule and zG layers. Red symbols indicate 280 mOsm; black symbols indicate 310 mOsm. Statistical significance was assessed using one-way analysis of variance with Tukey multiple comparisons test. **P less than .01; ns, not significant.

Finally, we hypothesize that targeting volume-regulatory mechanisms, such as the Na^2+^-K ^+^ -2Cl^−^ cotransporter 1 (NKCC1) to block osmolyte influx, could suppress Aldo production by lowering intracellular osmolarity (OSM_IC_), thereby increasing the osmotic gradient across the zG cell membrane, analogous to raising OSM_EC_. NKCC1 facilitates the electroneutral import of osmolytes (Na^+^, K^+^, Cl^−^) that drive water influx (37-39) and is highly expressed in mouse zG cells (21). Here, we confirm that *Slc12a2* (NKCC1, magenta) is expressed throughout the mouse adrenal, with particularly high expression in *Cyp11b2*+ (green) cells (Fig. 6A). In contrast, *Pecam1* (PECAM-1, magenta), a marker of endothelial cells, is expressed only in *Cyp11b2*- (red) cells (Fig. 6B). Next, we tested if blocking NKCC1 could suppress Aldo autonomy. We measured Aldo output from WT adrenal slices treated with TIs in either permissive (280 mOsm) or suppressive (310 mOsm) media, with or without 20 µM furosemide, an NKCC1 inhibitor. TI-evoked Aldo production was significantly reduced in slices treated with furosemide in 280 mOsm media (Fig. 6C, left). Notably, when slices were maintained in 310 mOsm media, where TI-induced stimulation is already minimal, furosemide failed to suppress basal Aldo production (see Fig. 6C, right). These findings demonstrate that limiting ion influx into zG cells can serve as a molecular brake on Aldo production.

## Discussion

Aldo production that is no longer regulated by the RAS underlies PA. Its prevalence is substantial and contributes significantly to the progression of hypertensive cardiovascular and renal diseases. While considerable research has focused on defining the causes of dysregulated Aldo synthesis, comparatively less attention has been directed toward identifying mechanisms capable of suppressing this aberrant production. In this study, we address this gap by investigating whether the osmolar-volume regulatory axis can serve as a potent attenuator of Aldo secretion and intracellular Ca^2+^ signaling both in acute (pharmacological) and chronic (genetic) models of PA. Using adrenal-slice preparations from TASK channel loss-of-function mouse models of PA, we demonstrate that increasing OSM_EC_ proportionally suppresses autonomous Aldo production by attenuating its primary intracellular signal, Ca^2+^ oscillatory activity. Furthermore, we corroborate and extend previous findings (11, 24), demonstrating that increased OSM_EC_ attenuates AngII-induced activation of zG rosettes in part through a novel signaling mechanism—specifically, by reducing Ca^2+^ burst duration. Our results suggest that the rosette architecture of the zG layer is intrinsically responsive to osmotic gradients, which can restrain zG excitability and steroidogenic output.

PA is characterized by several subtypes: Aldo-producing adenoma, Aldo-producing micronodules associated with bilateral adrenal hyperplasia, and familial hyperaldosteronism (FH), each with diverse underlying molecular etiologies. Effective control of Aldo autonomy in PA is a considerable clinical challenge. Next-generation sequencing has identified somatic and germline mutations in Aldo-producing adenoma and FH, respectively. Many of these genes (*KCNJ5* (40), *ATPA1* (41, 42), *ATP2B3* (41), *CACNAID* (42, 43), *CACNA1H* (22, 44), *CLCN2* (17, 18, 21), *MCOLN3* (45), *SLC30A1* (26)) modify how the zG cell controls intracellular Ca^2+^ (3). In addition, somatic mutations in *CACNA1D*, the gene that encodes the α-1D subunit of a voltage-dependent L-type Ca^2+^ channel, have been identified as the most common drivers of Aldo autonomy in micronodules associated with bilateral adrenal hyperplasia (43, 46). Thus, a comprehensive analysis of how Ca^2+^ signaling in the zG cell is controlled within the structural context of the rosette may offer a mutation-agnostic strategy for addressing the spectrum of Aldo dysregulation in PA. The present study builds on our previously published findings (9, 10) showing that: (1) zG cells in their native rosette organization are conditional oscillators; (2) AngII induces excitability by triggering bursts of large-amplitude Ca^2+^ oscillations, the frequency of which is proportional to steroidogenic output; (3) periodicity in zG cells remains AngII dose invariant, as does mean burst duration, unlike many neuronal voltage oscillators; and (4) zG responses in the loss-of-function TASK channel PA mouse model replicate those evoked by high-dose AngII. Using genetically defined mouse models and high-sensitivity Ca^2+^ imaging, we now show how OSM_EC_ alters the dynamics of Ca^2+^ signaling in the zG rosette. Specifically, elevated OSM_EC_ counteracts the actions of AngII-stimulated and TASK-induced autonomy, reducing both the number of active cells and the frequency of bursting, which is governed by burst initiation mechanisms. In addition, OSM_EC_ uniquely regulates burst termination, suggesting that OSM_EC_ targets at least one effector distinct from those maintaining zG-stimulation. Notably, we find that modulatory effects of osmolarity are consistent over time and reversible within individual slices, supporting a direct and dynamic role for osmotic regulation of zG cell function.

We used OSM_EC_ as an investigative tool to demonstrate that osmotic-regulatory mechanisms can suppress autonomous Aldo production in a model of PA. Although patients with PA often exhibit volume expansion, there is no evidence that their plasma osmolarity deviates from the normal physiological range. Moreover, therapeutically increasing plasma osmolarity to suppress Aldo production would be neither practical nor safe. However, the osmolar-volume regulatory axis of cells responds to the osmotic gradient determined by both OSM_EC_ and OSM_IC_, as well as membrane hydraulic conductivity and “spare” membrane folds (47). Our data indicate that increasing the OSM_EC_:OSM_IC_ ratio can suppress Aldo production; thus, we predict that lowering OSM_IC_ by either an increase in net osmolyte efflux or decrease in net osmolyte influx will have comparable outcomes to raising OSM_EC._ Notably, large fluctuations of OSM_IC_ occur both acutely, during periods of intense cellular activity as intracellular osmolyte accumulate (47), and daily, as a result of circadian time-dependent oscillations in soluble cytosolic protein expression in cells expressing clock genes (48), such as zG cells (49). Even small changes in intracellular macromolecule concentrations mediated by normal circadian and metabolic activity require corrective volume responses (48, 50). Thus, cells constitutively regulate their cell volume even under isotonic conditions, using the same molecular toolkit as in anisotonic stress to maintain cell volume homeostasis.

As proof of principle that targeting volume regulatory machinery to increase efflux or decrease influx of intracellular osmolytes can suppress autonomous Aldo under isotonic OSM_EC_, we pharmacologically blocked NKCC1, an osmolarity-sensitive cotransporter highly expressed in *Cyp11b2*+ zG cells (21) (see Fig. 6A) that facilitates bulk, electroneutral influx of Na+, K^+^, and Cl^−^ ions. Evidence that NKCC1 inhibition can reduce OSM_IC_ was first demonstrated in ventricular myocytes, where NKCC1 inhibition resulted in a 13% to 18% reduction in cell volume in isotonic media (51). We therefore predicted that by reducing OSM_IC_, NKCC1 inhibition would increase the OSM_EC_:OSM_IC_ ratio and restrain autonomous Aldo output from adrenal slices. Indeed, incubation of zG slices with furosemide constrained TI-evoked stimulation of Aldo production (see Fig. 6C), to a level equivalent to that induced by 310 mOSm suppression.

We acknowledge that NKCC1 is certainly only one determinant of OSM_IC_ in zG cells. Changes in osmolality can occur through the osmolar efflux of ions with substantial intracellular concentrations, such as Cl^−^, K^+^, and organic molecules, provided their transmembrane movement is not restricted by an opposing electrochemical gradient (ie, osmolyte movement is electroneutral). Notably, intracellular Cl^−^ concentration is unusually high in zG cells (17) and, unlike intracellular K+, ionic transport in response to changes in osmolarity can significantly alter intracellular Cl^−^ concentration (52). This makes Cl^−^ transporters and channels compelling candidate substrates for osmolarity-induced Aldo modulation. zG cells functionally express several Cl⁻ channels, including ClC-2 (53), which intriguingly is activated by cell swelling. Nevertheless, gain-of-function mutations in CIC-2 (eg, R172Q) identified in FH type II are depolarizing and enhance, not attenuate, zG Ca^2+^ oscillatory activity and Aldo output (21). Thus Cl⁻ efflux driving volume regulation must be electrically coupled to unidirectional cation movement, likely by K⁺, to achieve volume homeostasis (54), and may attenuate changes in membrane polarization that would be predicted by movement of Cl^−^ alone. In addition, net Cl⁻ loss may also modulate zG cell Ca^2+^ dynamics indirectly by affecting processes such as pH regulation, cytoskeletal organization, and/or the activity of other ion channels/transporters via with-no-lysine (K) kinases (see (52) for review). Future studies that directly measure intracellular Cl⁻ dynamics in zG cells under varying osmotic conditions or NKCC1 inhibition would be highly informative in elucidating the role of Cl⁻ in osmotic control of autonomous Aldo production.

In conclusion, our findings establish the osmolar-volume regulatory axis as a powerful and broadly acting modulator of zG cell excitability and steroidogenic output. We identified how oscillatory Ca^2+^ dynamics, the primary driver of Aldo output, changes with osmolarity. We propose that targeting volume regulatory ion transporters to decrease osmolyte influx (as demonstrated with NKCC1 inhibition) or increase osmolyte efflux can restrain autonomous Aldo production, independent of underlying channel mutations. Harnessing this physiological osmolar-regulatory pathway to suppress renin-independent Aldo production may provide a strategy to expand the medical toolbox for PA that is complementary to the direct targeting of Aldo synthase by nonsteroidal Aldo synthase inhibitors currently under development.

## Data Availability

Some or all datasets generated during and/or analyzed during the current study are not publicly available but are available from the corresponding author on reasonable request.

## Current Affiliation

Mohamed Diagne is currently affiliated with the Department of Translational Dental Medicine, Boston University, Boston, MA, USA.

## Abbreviations

Aldo: aldosterone
AngII: angiotensin II
AT1R: angiotensin II type 1 receptor
Ca^2+^: calcium
Cl^−^: chloride
FH: familial hyperaldosteronism
K^+^: potassium
KO: knockout
Na^2+^: sodium
NKCC1: Na^2+^-K ^+^ -2Cl^−^ cotransporter 1
OSM_EC_: extracellular osmolarity
OSM_IC_: intracellular osmolarity
PA: primary aldosteronism
RAS: renin-angiotensin system
ROI: region of interest
TASK: TWIK-related acid-sensitive potassium
TI: TASK inhibitor
WT: wild-type
zG: zona glomerulosa
